# Single-cell transcriptome profiling of human HSCs during development: new insights into HSC ontogeny

**DOI:** 10.1038/s41392-022-01301-7

**Published:** 2023-02-06

**Authors:** Edgar Grinstein, Csaba Mahotka

**Affiliations:** 1 Department of Hematology, Oncology and Clinical Immunology, Medical Faculty, Heinrich Heine University, Düsseldorf, Germany; 2 Institute of Pathology, Medical Faculty, Heinrich Heine University, Düsseldorf, Germany

**Keywords:** Haematopoietic stem cells, Gene expression analysis

A study recently published in *Nature* reported a single-cell transcriptome map of human hematopoietic stem cells (HSCs) and a gene expression signature that distinguishes nascent HSCs from non-HSCs during prenatal development.^1^ This transcriptome map provides an important tool for further elucidation of human HSC ontogeny and could also serve as a guide for generation of transplantable HSCs ex vivo,^1^ to widen the therapeutic application of HSCs.

Multipotent HSCs have the capacity for self-renewal and differentiation to replenish blood cell lineages. Hematopoietic stem cell transplantation (HSCT) is the first successful stem cell transplantation therapy, and approximately 50.000 patients undergo HSCT annually worldwide. HSCs arise from the hemogenic endothelium through the process termed endothelial-to-hematopoietic transition (EHT) during embryogenesis. Monitoring human HSCs during ontogeny presents a significant challenge and the understanding of their precise origin and development is incomplete.^2^ In this context, we refer to a recent paper published in *Nature* by Calvanese et al. that reported a single-cell transcriptome map of human hematopoietic tissues during gestation.^1^ Furthermore, the authors established a gene signature that distinguishes HSCs from hematopoietic progenitor cells during prenatal development as well as a single-cell atlas encompassing gene expression profiles of human HSCs at different developmental stages. The authors rely on this molecular map in elaborating the human HSC ontogeny. Moreover, they present data suggesting that the transcriptome map of HSC development can provide useful information for generation of transplantable HSCs from pluripotent stem cells (PSCs) ex vivo,^1^ to broaden the therapeutic use of HSCs.

**Fig. 1:**
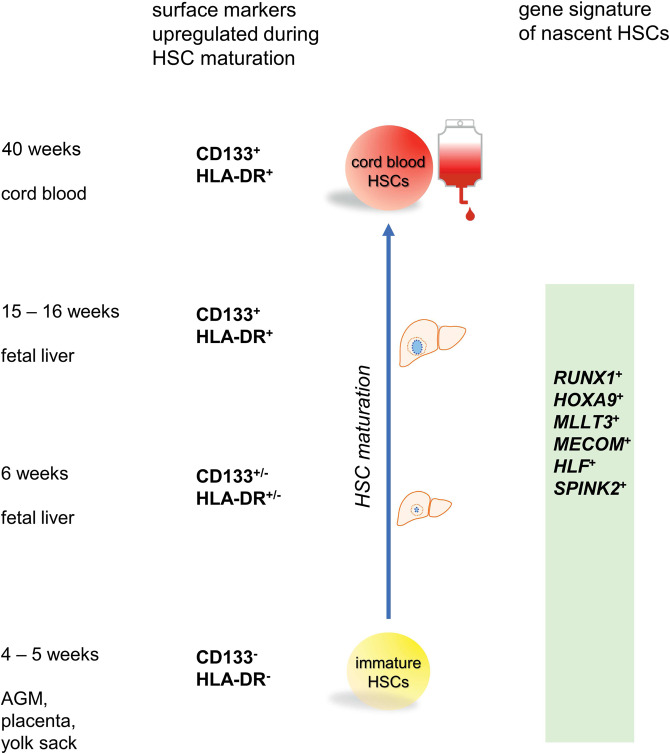
Landmarks distinguish HSCs and their developmental maturation during ontogeny. Surface markers CD133 and HLA-DR are upregulated during the developmental maturation of HSCs. Gene signature of highly enriched HSC genes identifies nascent HSCs as opposed to non-HSCs

In their study, Calvanese et al. conducted single-cell RNA sequencing (scRNA-seq) that is used for investigation of the global transcriptomic profile of a single cell, on CD34^+^ and/or CD31^+^ hematovascular cells from aorta-gonad-mesonephros (AGM) region. Analysis of cell type specific expression clusters revealed genes that are significantly enriched in HSCs, compared with other hematopoietic cells, thus allowing the authors to create a gene expression scorecard of nascent human HSCs.^1^ Furthermore, a scRNA-seq map of human hematopoietic tissues at different developmental stages was reported. The authors found that the signature of highly enriched HSC genes *RUNX1*^+^*HOXA9*^+^*MLLT3*^+^*MECOM*^+^*HLF*^+^*SPINK2*^+^ distinguishes nascent HSCs from non-HSCs during ontogeny, at anatomic sites that include the AGM region, placenta, yolk sack and fetal liver^1^ (Fig. 1). Comparison of gene expression profiles of HSCs at different maturation stages revealed that 20 established transcriptional regulators were expressed in these cells already after the HSC emergence. However, changes in gene expression associated with maturation of HSCs were detected and elaborated in the paper as the HSC maturation scorecard.^1^ Among the genes downregulated during HSC maturation were those associated with fetal properties and cell proliferation as well as genes encoding cell surface molecules CDH5, ITGA2B, IL3RA, and CSF1R. On the other hand, a marker of hematopoietic stem and progenitor cells CD133, which was reported as associated with specific events of cellular signaling in these cells,^3^ was upregulated in the course of HSC maturation as was also HLA-DR (Fig. 1), thus suggesting that HSC surface phenotype evolved during the development.^1^

Furthermore, the authors also elaborated molecular programs and cell populations that participate in the emergence of HSCs. They created an endothelial-to-hematopoietic transition scorecard encompassing human genes that serve as EHT landmarks and also other ones significantly regulated in the course of EHT.^1^ Moreover, analysis of the AGM region during the developmental window of HSC formation revealed that human HSCs originate from *ALDH1A1*^+^*KCNK17*^+^*RUNX1*^+^ hemogenic endothelial cells. These HSC precursor cells are preceded by *IL33*^+^*ALDH1A1*^+^ arterial endothelial cell population. In addition, by means of spatial transcriptome analysis and immunofluorescence analysis, Calvanese et al. were able to visualize the emergence of HSCs in intra-aortic hematopoietic clusters,^1^ in line with previous findings.^2^

Hematopoietic stem cell transplantation has been successfully used for treatment of certain life-threatening diseases for decades. However, its therapeutic use is often limited by obstacles including an inadequate availability of transplantable and immunologically compatible, healthy HSCs.^4^ In this context, de novo generation of HSCs from PSCs harbors therapeutic potential as an option to overcome this limitation.^4^ However, derivation of fully functional HSCs from PSCs presents a significant challenge, since the process of HSC generation is incompletely understood and therefore difficult to recapitulate ex vivo.^2^ On the basis of the molecular map of human HSC development, Calvanese et al. were able to assign HSPCs, derived from PSCs ex vivo, to their in vivo counterparts.^1^ Thus, the single-cell transcriptome map of HSC ontogeny is potentially useful as a guide for ex vivo generation of transplantable HSCs. The reported molecular map of human hematopoietic tissues during gestation^1^ can also increase our knowledge of certain prenatally initiated diseases, including pediatric leukemia.^5^

The study by Calvanese et al.^1^ provides an important contribution to the hematopoietic field by presenting new insights into the ontogeny of HSCs. HSCs emerge during embryogenesis and are the foundation for hematopoiesis. The study informs about the molecular identity, the precise origin, and the developmental maturation of nascent HSCs. The paper creates new perspectives for improved understanding of the etiology of congenital blood disorders, which is of relevance for development of new treatments, and an in-depth comment on this interesting aspect would be helpful. Future research will reveal the implications of the herein-reported knowledge for generation of transplantable HSCs ex vivo as well as for deciphering diseases initiated prenatally.
